# The Behavior of Industrial Wastes as a Replacement for Metakaolin Before Geopolymerization: A Comparative Study

**DOI:** 10.3390/ma18174035

**Published:** 2025-08-28

**Authors:** Michelina Catauro, Antonio D’Angelo, Francesco Genua, Mattia Giovini, José Miguel Silva Ferraz, Stefano Vecchio Ciprioti

**Affiliations:** 1 Department of Engineering, University of Campania “Luigi Vanvitelli”, Via Roma 29, 81031 Aversa, Italy; michelina.catauro@unicampania.it; 2 Department of Engineering “Enzo Ferrari”, University of Modena and Reggio Emilia, Via P. Vivarelli 10, 41125 Modena, Italy; francesco.genua@unimore.it (F.G.); mattia.giovini@unimore.it (M.G.); 3 Department of Basic and Applied Science for Engineering (S.B.A.I.), Sapienza University of Rome, Via del Castro Laurenziano 7, Building RM017, 00161 Rome, Italy; josemiguel.silvaferraz@uniroma1.it (J.M.S.F.); stefano.vecchio@uniroma1.it (S.V.C.)

**Keywords:** MK-based geopolymer, waste recycling, infrared spectroscopy, thermal stability, boiling and integrity tests, compressive strength, leaching test, antimicrobial activity

## Abstract

Today, several conventional wastes (fly ash, ground granulated blast furnace slags, etc.) are used as valid precursors for geopolymer synthesis. However, there are several new wastes that can be studied to replace geopolymer precursors. This study investigates the behavior of four industrial wastes—suction dust (SW1), red mud (SW2), electro-filter dust (SW3), and extraction sludge (SW4)—as 20 wt.% substitutes for metakaolin in geopolymer synthesis. The objective is to assess how their incorporation before alkali activation affects the structural, thermal, mechanical, chemical, and antimicrobial properties of the resulting geopolymers, namely GPSW1–4. FT-IR analysis confirmed successful geopolymerization in all samples (the main Si-O-T band underwent redshift, confirming Al incorporation in geopolymer structures after alkaline activation), and stability tests revealed that none of the GPSW1–4 samples disintegrated under thermal or water stress. However, GPSW3 showed an increase in efflorescence phenomena after these tests. Moreover, compressive strength was reduced across all waste-containing geopolymers (from 22.0 MPa for GP to 12.6 MPa for GPSW4 and values lower than 8.1 MPa for GPSW1–3), while leaching tests showed that GPSW1 and GPSW4 released antimony (127.5 and 0.128 ppm, respectively) above the legal limits for landfill disposal (0.07 ppm). Thermal analysis indicated that waste composition influenced dehydration and decomposition behavior. The antimicrobial activity of waste-based geopolymers was observed against *E. coli*, while *E. faecalis* showed stronger resistance. Overall, considering leaching properties, SW2 and SW3 were properly entrapped in the GP structure, but showed lower mechanical properties. However, their antimicrobial activity could be useful for surface coating applications. Regarding GPSW1 and GPSW4, the former needs some treatment before incorporation, since Sb is not stable, while the latter, showing a good compressive strength, higher thermal stability, and leaching Sb value not far from the legal limit, could be used for the inner reinforcement of building materials.

## 1. Introduction

Today, waste valorization and the need for recycling, minimizing source depletion, and implementing sustainable production play important roles in every economic sector. For example, organic wastes are valorized by extracting biomolecules feeding animals and producing bioenergy, while inorganic wastes are valorized through several processes (such as thermochemical and biochemical techniques and mechanical and chemical recycling). Among them, urban mining waste and industrial wastes are chemically treated to obtain eco-friendly building materials such as geopolymers and alkali-activated materials [1,2,3,4]. Geopolymers (GPs) and, in general, alkali-activated materials (AAM) are widely used because of their ability to entrap several wastes as precursors or fillers, obtaining materials useful for buildings, supplementary cements, and wastewater treatments [5,6,7,8,9], meeting the goals of the Circular Economy [10]. These materials are the main competitors of Ordinary Portland Cement (OPC), the production of which is highly polluting, since the need for high calcination temperatures leads to a huge amount of greenhouse gas emissions, as well as a huge amount of energy consumption [11,12]. GPs are a subclass of AAMs rich in Al and poor in Ca content that are activated in alkaline solutions (such as KOH, NaOH, sodium silicate, and potassium silicate) [13,14]. During this reaction, the aluminosilicate precursor (e.g., metakaolin or MK) undergoes dissolution, leading to the formation of inorganic oligomers composed of several Si^4+^ and Al^3+^ ions linked by bridging oxygen atoms originating from terminal OH groups. This is followed by polycondensation and reorganization, thus resulting in an amorphous material with a high mechanical performance [15,16,17]. Today, there are several wastes that can be used as geopolymer precursors (fly ash, biomass ash, mine tailings, ground granulated blast furnace slag, ceramic waste, red mud, etc.) [18]. Fly ash with a low Ca content has been widely used in geopolymers. Recently, Ren et al., 2024, investigated the effect of Ca content in FA-based geopolymers, concluding that a lower CaO content results in an increase in mechanical properties, while a higher CaO content leads to lower heavy metal leaching [19]. Banana peel ash and rice ash are examples of biomass ash used in geopolymer materials. It has been demonstrated that using 10 wt.% of banana peel powder strengthens halloysite-based geopolymer mechanical properties (up to 45 MPa), while supplying an alkali source [20]. Furthermore, substituting ground granulated blast furnace slag for the MK precursor led to an increase in early compressive strength and a decrease in compressive strength at 28 days of aging [21]. In a case study reported in [22], red-mud-based geopolymers were used to build a pavilion located in Aspra Spitia, Greece. A combination of 90 wt.% of fly ash and 10 wt.% of red mud led to a geopolymer product with a high mechanical strength after 28 d of aging (47.6 Mpa) [23]. Red-mud-based geopolymers possess an increased stability and higher mechanical properties when rice husk ash is added to the formulation [24]. In a case study reported in the literature, ceramic wastes from tiles, tuff, and porcelain showed good adhesion with MK-based geopolymers, resulting in colored geopolymers useful for cultural heritage [25]. It is worth nothing that the use of wastes in geopolymers causes non-homogeneous properties because of contaminants. On the other hand, the non-homogeneous properties of MK, caused by remarkable differences in structure, chemical composition, and reactivity, have been overcome in the past by synthesizing Al_2_O_3_·2SiO_2_ ceramic materials as precursors to replace MK in the preparation of geopolymers [26]. Since many wastes can be used either as a precursor or as filler, the final consolidated materials can show several properties [27,28,29]. Still, there is a need to understand how the presence of different wastes affects the properties of GPs. Moreover, the comprehension of waste behavior before and after alkaline activation is still an important aspect to consider, especially when new types of waste are being studied for the first time. Indeed, the novelty of this manuscript is due to the investigation of new types (except red sludge) of solid industrial wastes (SWs) replacing 20 wt.% of MK. The rationale behind this choice is related to the fact that these wastes have only been used as 20 wt.% of filler, and are, thus, added only after the alkali activation of MK [30]. In this study, the following four types of SW were used: (i) suction dust (SW1); (ii) red sludge from alumina production (SW2); (iii) electro-filter dust (SW3); and (iv) extraction sludge from partially stabilized industrial waste (SW4). The properties of consolidated geopolymers were investigated through Fourier-Transform Infrared spectroscopy (FT-IR), simultaneous thermal analysis, integrity tests, boiling tests, mechanical properties, leaching tests, and antimicrobial properties. Furthermore, since these wastes have been already used as filler at 20 wt.%, a comparison with this case study is reported in the Discussion Section 4.

## 2. Materials and Methods

### 2.1. Materials

Metakaolin (MK), purchased from IMCD Deutschland GmbH & Co., Cologne, Germany, is used as a main precursor for geopolymer synthesis. This MK is characterized by a d_50_ = 3.6 µm, a surface area via B.E.T. of 12 m^2^/g, and the following chemical composition: 53 wt.% SiO_2_, 40.5 wt.% Al_2_O_3_, 5 wt.% TiO_2_, and 1.5 wt.% of minor oxides [31].

**Figure 1:**
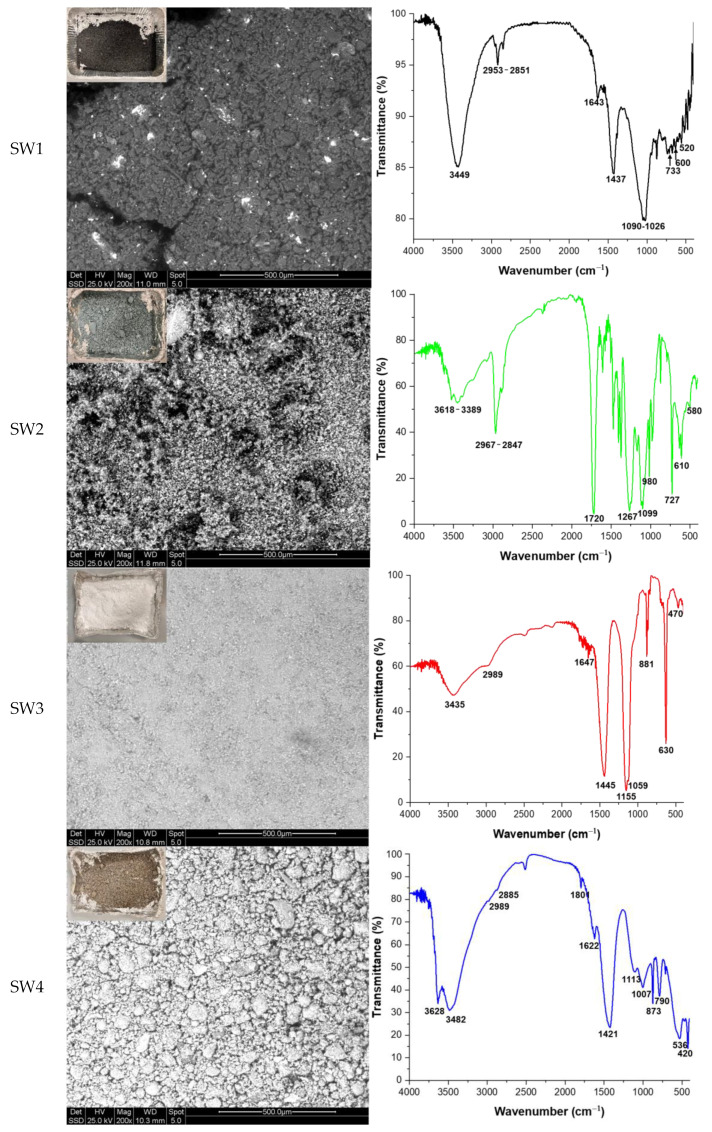
FT-IR spectra and macroscopic and microscopic images of wastes.

Four different types of industrial waste were provided by an Italian company located in Aversa. These waste materials, along with their main properties and labels, are detailed in Table 1, while their macroscopic and microscopic appearance and complex FT-IR spectra are shown in Figure 1. According to the data reported in Table 1, these wastes are characterized by the presence of heavy metals (As, Sb, Sn, Pb, Fe, Ni, Cr, and Cu—for all SW1–4), as well as chlorides (SW2 and SW3), sulfates (SW3), fluorides (SW3), and hydrocarbons of short and long chains (for all SW1–SW4). The presence of these contaminants disallows their disposal in common landfill, as they need to be treated and made inert before their disposal.

From Scanning Electron Microscopy (SEM) images, it can be observed that SW1 is composed of polyhedral spheres with a particle size in the range of 50–150 µm. Smaller powdery-like irregular particles are composed mainly of Sb (which agrees with data reported in Table 1). SW2 is composed of very fine particles, spherical in shape, with two different compositions and sizes: larger ones (10–20 µm in size) are composed of lighter elements and appear gray in color, while smaller ones (1–5 µm in size) are composed of heavier elements and appear white in color. SW3 shows particle sizes very similar to those of sample SW2, while SW4 is composed of large grains (about 200 µm) with an irregular shape, closer to that of rigid aggregates rather than to a single crystal. They are very homogeneous in the gray scale, indicating uniformity in chemical composition.

The FT-IR spectrum of SW1 shows –OH stretching and bending signals at 3449 and 1643 cm^−1^ [32], –CH_2_ and –CH_3_ vibration bands (2953–2851 cm^−1^ [32]) indicating organic matter, and carbonate vibrations (1437–1385 cm^−1^). In the fingerprint region (800–400 cm^−1^), there are transmittance bands of heavy metal oxides: peaks at 733 and 600 cm^−1^ are assigned to Sb–O–Sb [33] or Sn–O [34], and those at 520 cm^−1^ are assigned to Sn–OH. The FT-IR spectrum of SW2 displays numerous bands: the one at 2967–2847 cm^−1^ is assigned to C–H of short hydrocarbon chains [35], while the band at 1720 cm^−1^ is due to C–O vibration [32]. The peaks at 1099, 980, and 610 cm^−1^ could be associated with the presence of sulfates [36], while the ones located at 1267 cm^−1^ and 874 cm^−1^ are assigned to nitrates [37]. The peak at 727 cm^−1^ could be related to the presence of chlorine, whereas the peak at 580 cm^−1^ is related to Fe–O or Al–O [35]. SW3 exhibits transmittance bands at 3435 and 1647 cm^−1^ (–OH and H–O–H vibrations), 2989 cm^−1^ (C–H vibration due to the organic phase), 1059 and 881 cm^−1^ (O–S–O and O=S=O, from sulfates), 1445 cm^−1^ (carbonates [38]), and signals from 881 to 470 cm^−1^ related to metal oxides (Cu–O and Zn–O [39]). SW4 shows peaks at 3628 cm^−1^ (–OH bound to Sn, confirmed by 1113 cm^−1^ [35]), 3482 and 1622 cm^−1^ (H–O–H), 2982–2885 and 1421 cm^−1^ (C–H), 873 cm^−1^ (sulfates [35]), and 790–420 cm^−1^ (Zn–O, Mn–O, and V–O [39,40,41]).

Prior to their use, all wastes were ground and sieved to ensure that all particles possessed diameters smaller than 75 μm, ensuring uniformity for incorporation into the geopolymer formulations.

Sodium silicate solution, with a pH of 12.5 and a molar ratio of SiO_2_ to Na_2_O of 2.6, was provided by Prochin S.r.l., located in Caserta, Italy. Its chemical composition included 27.10 wt.% SiO_2_, 8.85 wt.% Na_2_O, and 64.05 wt.% H_2_O.

Sodium hydroxide pellet, MilliQ water, potassium bromide, and sodium chloride (reagent grade) were purchased from Sigma Aldrich in Milan, Italy.

Tripton Bile X-gluconoside Agar medium and Slanetz Bartley medium were purchased from Liofilchem S.r.l., Roseto degli Abruzzi, Italy. *Escherichia coli* (ATCC 25922) and *Enterococcus faecalis* (ATCC 29212) were purchased from VWR International Eurolab SL, Barcelona, Spain.

### 2.2. Methods

**Figure 2:**
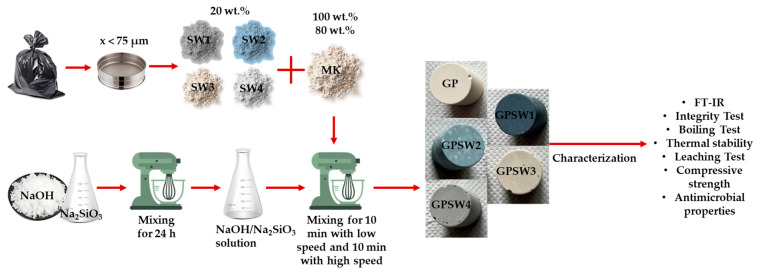
Flowchart procedure for geopolymer synthesis.

The mixing process (the flowchart procedure of which is shown in Figure 2) consisted of mixing the dried MK powder with the activating solution at low speed for 10 min and at high speed for another 10 min. Geopolymer samples containing waste were prepared by mixing 20 wt.% of SW1, SW2, SW3, and SW4 with 80 wt.% of MK prior to mixing with the activating solution (all formulations are reported in Table 2). The GP0 and GPSW1–4 compositions were optimized according to the following ratios: SiO_2_/Al_2_O_3_ = 4, Na_2_O/Al_2_O_3_ = 1, and H_2_O/Al_2_O_3_ = 13, with the slight differences in these ratios due to the waste compositions. The synthesis of the GPs was performed using an AUCMA SM-1815Z electric mixer (AUCMA Co., Ltd., Qingdao, China). After the mixing procedure, the fresh GP pastes were sealed in plastic molds and cured for 24 h in an oven at 25 °C. After curing, the samples were demolded and allowed to age at room temperature for 7, 14, and 28 days.

FT-IR analysis was carried out with the Prestige21 Shimadzu system (Shimadzu Italia S.R.L., Milan, Italy), equipped with a DTGS KBr detector (Shimadzu Italia S.R.L., Milan, Italy). A resolution of 2 cm^−1^ and 60 scans were used. The spectra were acquired within the range of 400–4000 cm^−1^ by analyzing KBr disks (2 mg sample and 198 mg KBr). The FT-IR spectra were processed using IRsolution (v.160, Shimadzu, Milan, Italy) and Origin 8 (v.2022b, OriginLab Corporation, Northampton, MA, USA) software.

Morphology observations were conducted on manually tapped powders from the industrial wastes on carbon tape by ESEM (Environmental Scanning electron microscopy) using a QUANTA 200 (FEI, Eindhoven, The Netherlands) microscope in high vacuum mode. The backscatter (BSE) detector was used for atomic number contrast images and the Oxford–Link Inca (Oxford, UK) 350 X-ray spectrometer for chemical analysis.

The integrity test was performed by soaking the geopolymers in MilliQ water (at a mass-to-volume ratio of 1 g/100 mL) for 24 h, following the protocol reported in [42]. After 24 h of immersion, the integrity of the samples was estimated in terms of visible fractures and visible fragments in water leachates, as well as mass gain or loss after test [42]. Water leachates from integrity tests were used to measure Ionic Conductivity (IC) and pH by Crison GLP31 and Crison GLP21, Hach Lange Spain, S.L.U, Barcelona, Spain.

The boiling water test was performed according to [43,44] by keeping the samples in boiling water for 20 min and performing a rapid durability assessment evaluated through their qualitative structural resistance.

Simultaneous thermogravimetry–differential thermal analysis (TG-DTA) was performed using a simultaneous TG/DTA apparatus (Stanton-Redcroft 1500, Copper Mill Lane, London, UK). Experiments were carried out under an argon flow rate of 40 cm^3^·min^−1^ to observe the sample thermal behavior [45]. The heating rate used was 10 °C·min^−1^. Open-pan platinum crucibles were used for the TG/DTA experiments, with each experiment employing 6 to 10 mg of the different samples. The system was calibrated using several high-purity standards, including tin and indium [46], tailored to the specific temperature range under investigation.

The compressive strength was measured by using a Dual Column Testing System (INSTRON, series 5967-INSTRON, Norwood, MA, USA) configured with a crosshead speed of 1 mm/min. The test was conducted with 5 specimens (cylindrical molds −27 mm × 54 mm) per each geopolymer type.

Leaching tests on samples aged for 28 days were performed according to EN 12457-2:2004 [47]. Specifically, 5 g of the material was ground and sieved to a particle size of less than 2 mm, then placed in Teflon^®^ containers. These samples were immersed in distilled water using a 1:10 solid-to-liquid mass ratio and stirred magnetically for 24 h to ensure thorough interaction between the solid and the water. After the test, the resulting leachates were separated from the solid residues, acidified to a pH of 2 using a nitric acid solution, and analyzed using inductively coupled plasma mass spectrometry (ICP-MS). For this analysis, an iCAP TQ ICP-MS spectrometer (Thermo Fisher Scientific Inc., Waltham, MA, USA) was used to measure the ion concentrations in the diluted leachate samples. These dilution factors were accounted for when calculating the original leachate concentrations.

The antimicrobial activity of the material samples was evaluated using the Kirby–Bauer disk diffusion method [48]. Two bacterial strains, *E. coli* and *E. faecalis*, were selected because of their correlation to polluted environments and infection [49]. Prior to testing, the samples were ground into powder and compressed into discs weighing 200 mg, then sterilized using UV light for one hour. TBX Medium and Slanertz–Bartley agar based-medium were prepared by autoclaving at 120 °C for 15 min and allowed to cool before being poured into Petri dishes at approximately 50 °C. The bacterial strains were suspended in a 0.9% NaCl solution to a concentration of 10^9^ CFU/mL and spread onto the appropriate solid media. Sample discs were placed at the center of each Petri dish prior to incubation. Incubation was carried out at 44 °C for *E. coli* and 36 °C for *E. faecalis* for 24 and 48 h [50]. After incubation, the inhibition halo diameters (IHDs) were measured. Three replicates were used to measure Mean ± Standard Deviation.

## 3. Results

### 3.1. Geopolymer Stability

**Figure 3:**
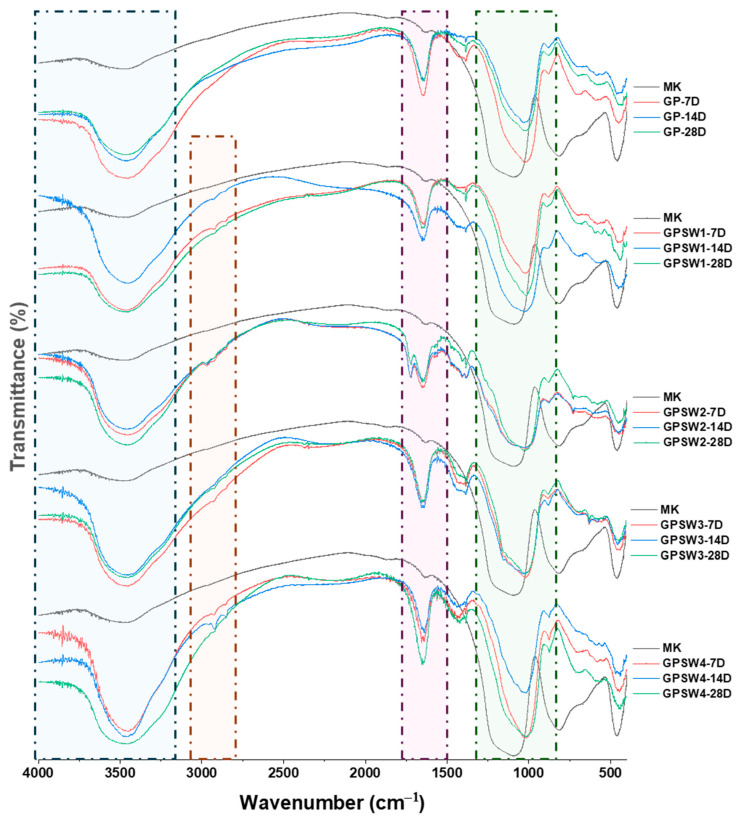
FT-IR comparison spectra of geopolymers at different aging times.

FT-IR analysis was performed to investigate the influence of wastes added to MK at 20 wt.% before alkali activation. FT-IR spectra were recorded at 7, 14, and 28 days of aging and are reported in Figure 3. Regarding the control, GP showed a characteristic redshift in the main absorption band (see the green square in Figure 3) centered at 1090 cm^−1^ in the MK precursor. This shift occurred because of the substitution of Si atoms with Al atoms, thus leading to a Si-O-Al bridge in the formed N-A-S-H (sodium aluminum silicate hydrate) gel, typical of a geopolymer network [51,52,53]. Indeed, for GP, this peak was centered at 1027 cm^−1^ for the 7- and 14-day aged samples and at 1019 cm^−1^ after 28 days of aging. GP spectra were also characterized by the presence of OH- stretching and bending (see the blue and violet squares in Figure 3) vibrations at 3470 and 1655 cm^−1^, thus indicating that water was released from the geopolymer samples during geopolymerization reactions, as well as water from the activator solution entrapped in the GP structures. Considering the GPSW1, GPSW2, GPSW3, and GPSW4 spectra during aging times of up to 28 days, there was a redshift for all of them, thus suggesting that mixing the wastes with the MK precursor before the addition of the alkali activator did not influence the geopolymerization reactions. Even though the geopolymer spectra containing wastes hid most of the waste IR peaks, slight differences in spectra were still appreciable. Indeed, the peaks in the range of 2980–2850 cm^−1^ were associated with C-H vibration modes (see the orange square in Figure 3) due to the organic contaminants in the wastes [32]. Moreover, GPSW1 spectra showed SW1 peaks in the finger printing region (from 800 to 400 cm^−1^) related to Sb-O and Sn-O vibrations. Furthermore, in the GPSW2 samples, the sharp band at 1720 cm^−1^ was due to the C-O vibration [35], while the strong sharp peak of SW3 located at 1445 cm^−1^ was still visible in GSW3 at different aging times, which is also related to carbonates.

**Figure 4:**
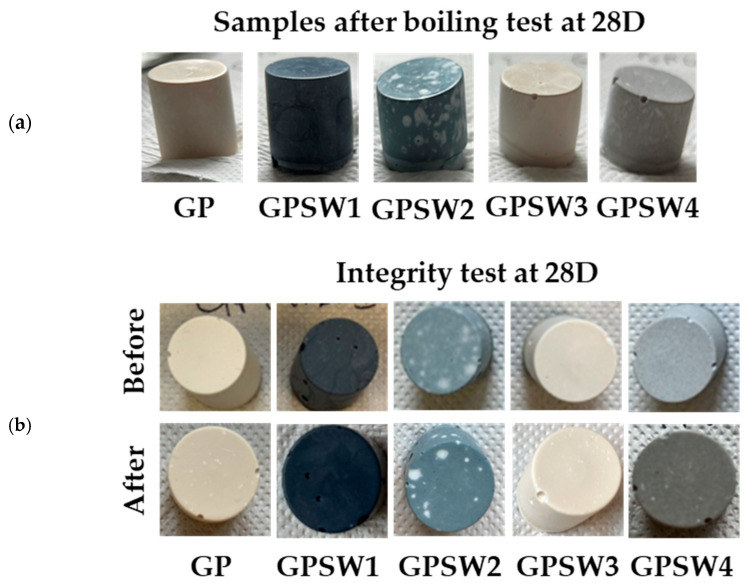
(**a**) Geopolymer samples after boiling tests at 28 days of aging. (**b**) Geopolymer samples before and after the integrity test at 28 days of aging.

To assess geopolymer stability from a macroscopic point of view, boiling and integrity tests were performed on all samples at different aging times. Images of both tests at 28 days are reported (Figure 4a and b, respectively). According to findings from the literature, a well-formed geopolymer can resist boiling water for 20 min [43]. In all tests performed at different aging times, none of the samples underwent disruption during the test, thus further confirming geopolymerization occurrences. This also indicates that the synthesized geopolymers are stable and long-lasting in harsh environments [54]. Moreover, none of the samples underwent disruption during and after integrity tests, additionally confirming macroscopic stability [55]. The white spots on the GPSW2 samples in both the integrity and boiling tests could be related to the efflorescence phenomenon as a consequence of imperfectly balanced charges at the molecular level, leading to this macroscopic side effect [56,57,58]. Indeed, when Na^+^ was not properly counterbalancing the Al(O_4_)^−^ in the GP structure and interacted with carbon dioxide from the atmosphere, this led to sodium carbonate formation on the surface. This is also confirmed by the presence of a sharp peak at 1720 cm^−1^ in GPSW2 assigned to C-O.

Geopolymer network stability was investigated through IC and pH measurements (summarized in Table 3) from water leachates of the integrity tests. According to the results obtained, GP reference showed a slight increase in IC values from 14 to 28 days of aging, maybe due to continuous network stabilization. In the reference sample, the IC depended mainly on Na^+^ and OH^−^ species released from the network [59,60]. GPSW1, GPSW2, and GPSW3 showed a decrease in IC values from 7 to 14 days of aging. Moreover, after 28 days, there was no variation in IC data, suggesting that the network was stable. Furthermore, GPSW3 showed very high IC values, maybe due to the possible presence of chlorides, fluorides, and sulfates, since SW3 is very rich in them. GPSW4 showed a lower IC value only after 28 days of aging, suggesting that the network was still under organization. It is worth noting that as the aging time increased, the IC values of the samples became closer to the values of GP (except the IC values of GPSW3, probably due to the high ionic content of SW3). Regarding the pH, all specimens showed a strong alkaline environment, since the pH values were all above 10.9.

### 3.2. Thermal Behavior and Thermal Stability

**Figure 5:**
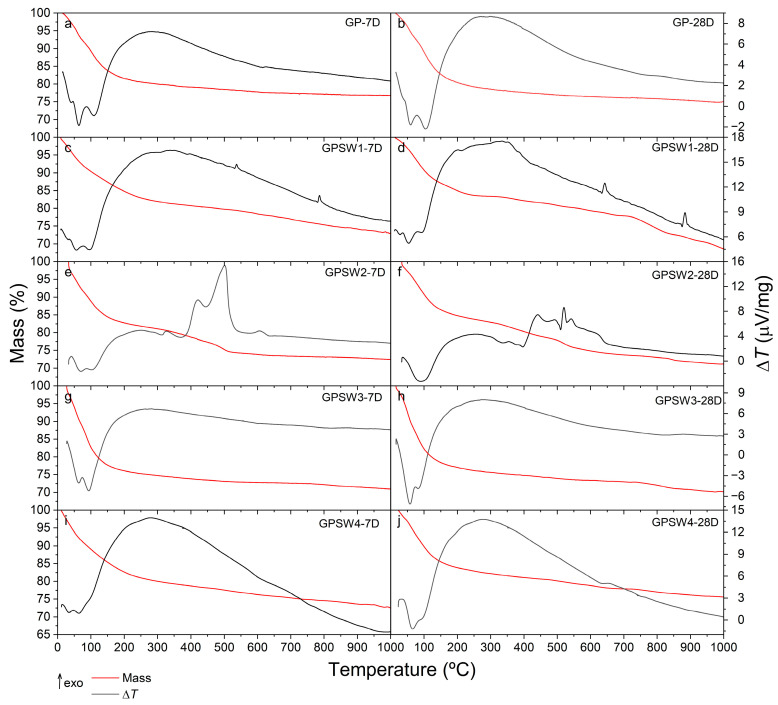
TG-DTA curves of the GP and GPSW(1–4) series obtained from several non-isothermal experiments from RT up to 1000 °C at a heating rate of 10 °C·min under an Ar flow of 40 cm^3^·min^−1^.

The simultaneous TG-DTA curves of GPs aged for either 7 or 28 days (denoted as 7D and 28D, respectively) are compared in Figure 5 (plots a and b, respectively) with those of the four GPs prepared with four different wastes (GSPW1, GPSW2, GPSW3, and GPSW4) under the same two aging conditions (plots c to j). The temperature ranges and total amounts of differently bound water contained in the materials (expressed as mass percentages) are summarized in Table 4 for comparison purposes.

Plots a and b of Figure 5 show that the GP sample undergoes two distinct mass loss steps, the first of which, up to about 270–280 °C, is ascribed to the loss of chemically and physically bound water, as shown by the presence of three poorly distinguished endothermic effects in the DTA curves. The comparison of the TG/DTA curves in plot a with those in plot b demonstrates that for the pristine GP, the differences are negligible and may be attributed to the aging time. A second step of mass loss takes place in a very wide temperature range (from 280 to about 700 °C) without remarkable endothermic or exothermic effects. Based on previous studies carried out on geopolymers under an inert atmosphere, this process may be reasonably attributed to dehydroxylation, which leads to a loss of water due to the condensation of hydroxyl surface groups. Similar conclusions can be drawn for GPSW3 and GPSW4.

The amount of water released by GPSW1-7D due to dehydration (plot c of Figure 5) is the lowest (around 17% by mass), and after 28 days of aging, this content is slightly lower (plot d of Figure 5). Similar conclusions can also be drawn from the analysis of GPSW2, where the percentage of water loss is even more noticeable (plots f and j of Figure 4).

In the case of GPSW1 (Figure 5 plots c and d), two exothermic peaks on the DTA signal in both samples (7 and 28D) are observed. The fact that these are not accompanied by a more significant mass loss (besides dihydroxylation, which is also observed in GP0) is an indicator that these are physical changes in the samples. The most likely case is that these are changes in the crystalline network of the sample. An additional experiment, i.e., a second heating scan of the sample, is performed. In this case, none of the peaks are observed, leading to the conclusion that these processes are irreversible [61,62]. Nevertheless, this sample seems to become less stable with time. In the GPSW1_28D sample, additional mass loss is observed between 700 and 850 °C, and the residual mass at the end is smaller.

For GPSW2 (Figure 5, plots e and f), several exothermic peaks are observed between circa 350 and 550 °C. As these signals are also accompanied by at least one identifiable mass loss, these processes can be ascribed to decomposition taking place in the sample. As this sample has a larger quantity of hydrocarbons, their thermal decomposition can lead to the exothermic event observed in the experiments. This is supported by the complex FT-IR print. Furthermore, at circa 650 °C, an exothermic, longer peak is observed on the DTA signal that is not accompanied by mass loss. This seems to be an irreversible relaxation of the geopolymer network.

Regardless of aging time, whether at 7 or 28 days (7D or 28D, respectively), the TG/DTA curves of both the GPSW3 (Figure 5, plots g and h) and GPSW4 (Figure 5, plots i and j) samples are very similar to that of the pristine GP, thus demonstrating that the waste composition of these two samples (Cu-rich GPSW3 with a very low amount of hydrocarbons and selenium-rich GPSW4 with a C10–C40 content lower than 100 ppm) does not affect their thermal behavior. However, it is important to note that, on the one hand, GPSW3 seems to lose stability with time as a new mass loss appears at circa 750 °C, but on the other hand, GPSW4’s stability seems to increase with time, as it has less water and the residual mass at the end is higher at 28D than at 7D.

**Figure 6:**
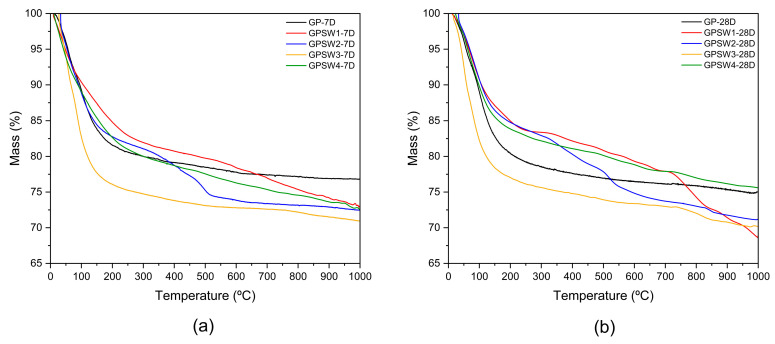
TG curves of the GPX series: (**a**) 7D vs. (**b**) 28D obtained using several non-isothermal experiments from RT up to 1000 °C at a heating rate of 10 °C/min under an Ar flow of 40 cm^3^/min.

Furthermore, a comparison of the different TG curves for the different samples at 7D and 28D is presented in Figure 6.

The relative thermal stability of the geopolymer samples can be evaluated based on their residual mass at the end of the TG experiments. At 7 days of aging (7D), most samples exhibit comparable residual masses (within about 1%), except for GPSW3, which shows the lowest stability—consistent with its higher initial water content (25.1%, Table 4). However, prolonged aging to 28 days (28D) reveals a significant divergence in stability among the samples (Figure 6b). The most pronounced differences are observed for GPSW1 and GPSW4, which exhibit opposing trends: GPSW1 undergoes a marked loss of stability over time, with its residual mass decreasing by nearly 5% at 28D compared to 7D. This degradation aligns with the appearance of an additional mass loss step between 700 and 850 °C in the TG curve (Figure 6), suggesting structural breakdown or further dehydroxylation at elevated temperatures. In contrast, GPSW4 demonstrates an enhanced stability upon aging, retaining a significantly higher residual mass at 28D. This improvement correlates with its reduced water content (17.5% at 28D vs. 20.0% at 7D, Table 4), implying that a slower dehydration rate or a more condensed geopolymer network may contribute to its durability.

The stability of GPSW2 and GPSW3 also evolves with aging, though less dramatically. GPSW3, despite its initial higher water content, shows a new mass loss at ~750 °C after 28D (Figure 6b), further underscoring its declining stability. These trends underscore the critical influences of waste composition (e.g., hydrocarbon content in GPSW2 and Se/Cu in GPSW4/GPSW3) and aging duration on the thermal resilience of geopolymers. Notably, GPSW4 emerges as the most stable formulation over time, likely due to its low volatile content and efficient geopolymerization.

### 3.3. Mechanical Behavior

**Figure 7:**
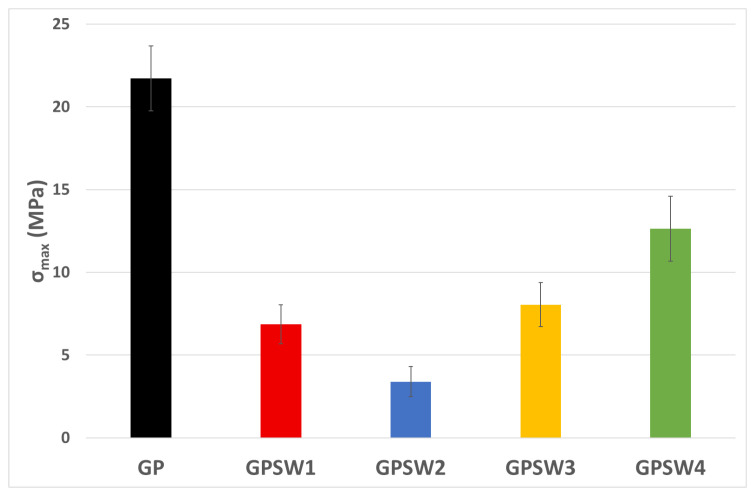
σ_max_ values of geopolymers after 28D.

The compressive strength results after 28 days of aging are reported in the histogram in Figure 7. The replacement of MK with industrial wastes led to a decrease in mechanical properties. Indeed, all σ_max_ values for the GPSW(1–4) series were lower than the reference one (22.0 MPa). This reduction in mechanical strength may have been due to the reduction in Al content in the GPSW(1–4), since 20 wt.% of MK was replaced with wastes [63,64,65], while the amount of activation solution was decreased according to the MK content. Specifically, for the GPSW1 sample, the Fe present in SW1 could replace Al, improving the compressive strength. However, the Ca and K present in this waste could also increase the positive ions, which would cause electrostatic repulsions at molecular level, compromising compressive strength [63]. This occurred even in the GPSW2 and GPSW3 samples, which were rich in chlorides and sulfates, even though the former had a high Al and Fe content. The higher compressive strength of GPSW4 with respect to the other waste-containing geopolymers could be related to its better-structured network, as also confirmed by thermal analysis.

### 3.4. Leaching Test

**Figure 8:**
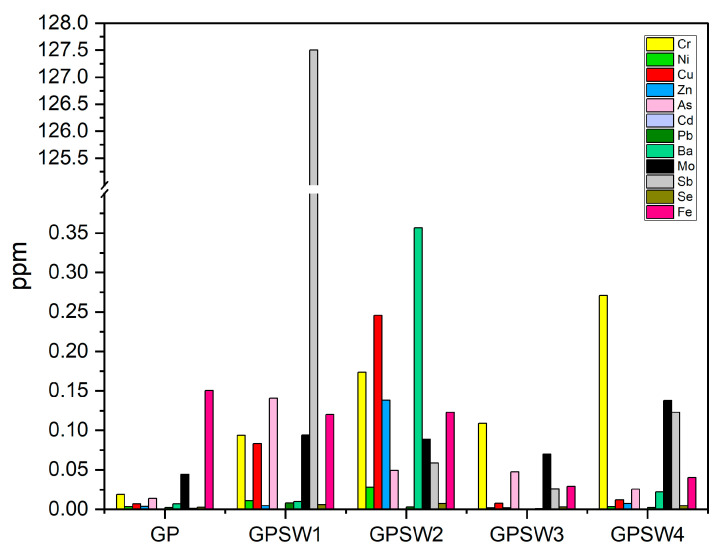
Heavy metal ions leached from GP samples after 28 days of aging.

The histogram in Figure 8 reports data on the leached heavy metals of samples aged for 28D. According to the legal limits for disposal in landfills based on the leachate (see Table 5), GPSW1 and GPSW4 released Sb ions above 0.07 ppm. In particular, GPSW1 released 127.4 ppm, while GPSW4 released 0.128 ppm. For this reason, the former can be considered hazardous in the case of landfill disposal, while the latter is in the range of non-hazardous and hazardous. Considering the data reported in Table 1 on heavy metals leached from the wastes, both SW1 and SW4 showed lower ppm values, meaning that the alkaline environment of the geopolymers increased the leaching rate of Sb, which possesses an amphoteric nature. Indeed, according to [66,67], in alkaline environments, Sb from SbO_3_H_3_ forms SbO_3_H_2_^−^, which is highly soluble, while Sb from Sb_3_O_6_OH leads to SbO_3_H_3_, which, in turn, forms SbO_3_H_2_^−^ again, contributing to an increase in Sb ions release. Regarding all the other geopolymer samples, none of them had hazardous heavy metal release, thus showing a stabler structure.

### 3.5. Antimicrobial Analysis

**Figure 9:**
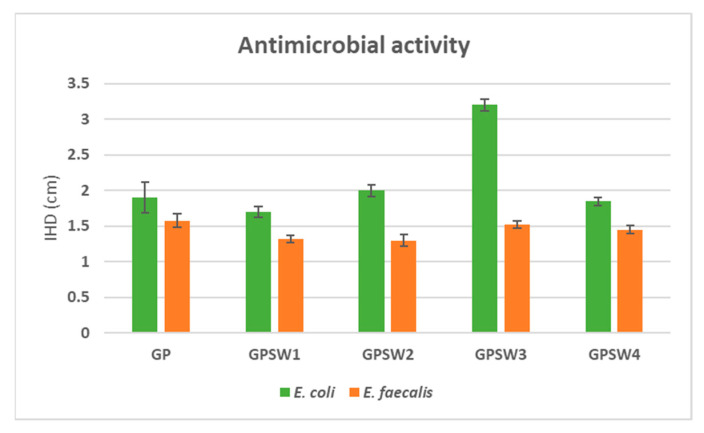
Inhibition halo diameters obtained after the growth of *E. coli* and *E. faecalis* in the presence of geopolymer samples aged for 28 days.

Figure 9 shows the antimicrobial properties of the geopolymer samples. All geopolymer samples had higher antimicrobial activity with respect to *E. coli*. Since most of the geopolymer samples (except GPSW1) showed no release of heavy metal ions in huge amounts, antimicrobial activity against this Gram-negative bacterium could be attributable to the alkaline environment. Indeed, according to [68], this bacterium undergoes failure in pH homeostasis and starts producing heat-shock protein, as well as inducing SOS, which leads to cell division inhibition. This antimicrobial property was also enhanced in the geopolymer containing SW3 (IHD value equal to 3.2 cm). This may be attributed to the high content of chlorides in this waste. Indeed, an increase in Cl^−^ in the cytosol of this bacterium leads to an increase in oxidative stress, which, in turn, causes cell death [69]. On the other hand, most of the results obtained for the treatment of *E. faecalis* with the geopolymers showed lower antimicrobial properties. This higher resistance to the alkaline environment is attributable to the fine regulation of homeostasis. Indeed, during this kind of environmental stress, this Gram-positive bacterium can synthesize membrane proteins which finely regulate H^+^/OH^−^ concentrations and increase its survival [70,71,72].

## 4. Discussion

The main aim of this study is understanding GP properties in the case of substituting 20 wt.% of MK precursor with four industrial wastes. In our previous work, the valorization of the same industrial wastes through their incorporation into metakaolin-based geopolymers was addressed [30]. However, both studies differ in strategy, analytical scope, and resulting material performance. In the previously cited study, industrial wastes were introduced as fillers to geopolymer fresh paste after alkali activation, while in this study, a more integrated approach was adopted, substituting 20 wt.% of metakaolin with waste before the geopolymerization process. This distinction significantly influenced the microstructure and performance of the final materials. It is worth noting that the FT-IR analyses in both cases confirmed geopolymer network formation via redshifts in the Si–O–T (T = Si or Al) stretching bands, yet the degree of shift and secondary peaks varied due to the interaction of the waste with the matrix at different synthesis stages. A comparison between data obtained at 28D in this study with those obtained in the previous one [30], at the same aging time, is reported in Table 6. Table 6 highlights that geopolymer samples with wastes are added before alkaline activation possess higher pH values and lower IC values, which could be related to higher stability [59]. Despite this, thermal analysis showed a higher stability for all samples in which these wastes were added as filler after the alkaline activation of MK. Indeed, the residues at 1000 °C were below 16%. According to the literature, a mass loss of about 16% up to 1000 °C is a good achievement for geopolymers [73,74]. In the previous study, a higher mass loss (up to 23%) for red-mud-based geopolymers (namely, 80GP20SW2) was consistent with that achieved by GPSW2 (28%), which also identified multiple decomposition stages. Regarding GPSW1, GPSW3, and GPSW4, all of them showed a higher weight loss, thus suggesting a lower thermal stability. From the mechanical properties point of view, adding the different wastes as fillers led to superior compressive strengths. Indeed, when suction dust was added after alkali activation, the resulting geopolymers reached σ_max_ = 58.5 MPa, while a lower value was achieved when adding SW1 before alkali activation (σ_max_ = 6.9 MPa), suggesting slower network consolidation when wastes were introduced pre-activation. These strong decreases in compressive strength were similar even for the other geopolymer samples. This suggests that these types of wastes are more useful as fillers than as precursors, especially to obtain material with a higher compressive strength. Leaching tests in both studies emphasized the importance of waste selection. Indeed, both geopolymer samples containing SW1 released hazardous antimony (Sb) levels. However, it seemed that the Sb concentration leached from 80GP20SW1 was 25 ppm, while in GPSW1, it was 127.5 ppm. Furthermore, when extraction sludge from the food supplement industry was added before alkaline activation, there was a slight increase in Sb release from GPSW4 (0.128 ppm), while it was not released when added as filler. Finally, both studies evaluated antibacterial activity and reported promising results against *E. coli*, attributing the effect to the alkaline pH and the presence of metal cations. In both cases, these enhanced antimicrobial activities make these types of geopolymers (except those containing SW1) useful for coating applications.

## 5. Conclusions

This study explored the substitution of 20 wt.% of metakaolin with four different industrial wastes (SW1–SW4) in geopolymer synthesis, evaluating the resulting materials in terms of structural, thermal, mechanical, chemical, and antimicrobial properties. The experimental findings demonstrated the following:

1. None of the wastes had a negative influence on geopolymerization, as confirmed by the redshift of the Si-O-T transmittance band in FT-IR spectra;
2. Macroscopically, all geopolymers showed structural stability, indeed, none of them underwent degradation during or after boiling and integrity tests. However, after these tests, GPSW2 showed huge white spots due to efflorescence phenomena;
3. Leaching water from integrity tests revealed an alkaline pH (10.9–11.8) for all samples, while the IC values were high for GSPW3 (12.7 mS/m after 28 days of aging);
4. Thermal analysis revealed that only GPSW4 showed a high thermal stability, with a mass loss of 17.5% at 28 days of aging;
5. Leaching tests underlined that geopolymers containing SW1 and SW4 released antimony (127.5 and 0.128 ppm, respectively) levels;
6. Antimicrobial properties revealed enhanced activity against *E. coli*, particularly in GPSW3 (IHD = 3.2 cm), but limited activity against *E. faecalis* due to its greater resistance to alkaline conditions.

In general, from the leaching results, SW2 and SW3 were properly entrapped in the GP structure, but showed lower mechanical properties. However, because of their antimicrobial activity, they could be useful for surface coating applications. Regarding GPSW1 and GPSW4, the former needs some treatment before incorporation, since Sb is not stable, while the latter, showing a good compressive strength, higher thermal stability, and leaching Sb value (0.128 ppm) not far from the legal limit (0.07 ppm), could be used for the inner reinforcement of building materials.

Overall, the incorporation of these industrial wastes before geopolymerization presents both opportunities and challenges. While enabling the valorization of waste streams and supporting Circular Economy goals, the careful selection and characterization of wastes are essential to ensure the environmental safety and functional performance of the resulting geopolymers.

## Figures and Tables

**Table 1:** Labels and waste characterization summary. All these data were provided by the company.

Type of Waste	Suction Dust	Red Mud ^a^	Electro-Filter Dust	Extraction Sludge ^b^
Sample label	SW1	SW2	SW3	SW4
Characteristics	Powdery, grey-black color	Powdery, grey-green color	Powdery, white color	Grainy, beige color
Residue (%) at 105 °C	99.1 ± 0.8	58.3 ± 2.0	98.6 ± 2.5	65.0 ± 3.7
pH	7.2	3.0	11.2	7.1
Chlorides (mg/L)	<5	25,678	25,980	-
Sulfates (mg/L)	<5	64.4	16,510	-
Fluorides (mg/L)	<5	0.35	3900	-
Nitrates (mg/L)	<5	17.5	-	-
Phosphates (mg/L)	<5	-	-	-
Cyanides (mg/L)	-	<50	-	-
Hydrocarbons (mg/L)	<100	C10–C40 < 2.5 C5–C8 = 294	C10–C40 < 18 C5–C8 < 1	C10–C40 < 100
Metal content (mg/kg)	As = 870 Sb = 145,265 Ca = 369 Fe = 219 Ni = 23 Pb = 228 K = 171 Sn = 257,741 Zn = 60	Al = 114 Cr = 130 Fe = 371 Mn = 371 Ni = 176 Pb = 25 Cu = 52 Zn = 25,426	As < 2.3 Cd < 2.3 Co = 1.35 Cr = 15.5 Mn = 15.5 Mn = 2.7 Mg = 43.2 Ni = 7.43 Pb = 21.5 Cu = 25.7 Sn = 7.4 Tl = 0.676 V = 1.35 Zn = 7.43	Sb < 10 As < 5 Cd < 0.6 Cr < 10 Mn = 22 Mg < 0.5 Ni = 2 Pb < 5 Cu < 5 Se < 100 V < 5 Zn = 25
Heavy metal leaching (mg/L)	Sb = 19.3	Cr = 0.2 Cu = 2.6 Ni = 7.37 Pb = 1.16 Zn = 168	As = 2.3	As < 0.01 Cd < 0.001 Cr = 0.01 Cu = 0.01 Mg < 0.0005 Ni = 0.01 Sb < 0.0005 Se < 0.01 Zn = 0.9 V < 0.02

^a^ from alumina production. ^b^ from food supplement industry.

**Table 2:** Geopolymer formulations.

Geopolymer Name	Mass/g						
	Na_2_SiO_3_	NaOH	MK	SW1	SW2	SW3	SW4
GP0	145.63	13.31	100.00	-	-	-	-
GPSW1	116.50	10.65	80.00	20.00	-	-	-
GPSW2	116.50	10.65	80.00	-	20.00	-	-
GPSW3	116.50	10.65	80.00	-	-	20.00	-
GPSW4	116.50	10.65	80.00	-	-	-	20.00

**Table 3:** IC and pH measurements from integrity test water leachates at different aging times. pH measurement error of ±0.1 pH and IC measurement error of ±0.2 mS/m.

Data from Integrity Tests	GP	GPSW1	GPSW2	GPSW3	GPSW4
IC (mS/m)-7D	2.2	4.4	4.5	17.5	4.6
IC (mS/m)-14D	2.0	2.1	3.3	12.6	4.4
IC (mS/m)-28D	2.8	2.2	3.3	12.7	3.2
pH-7D	10.9	10.9	11.0	11.0	10.9
pH-14D	11.2	11.5	11.7	11.8	11.6
pH-28D	10.9	10.9	11.3	11.3	11.0

**Table 4:** Water content analysis of the GP and GPSW (1-4) series (7D vs. 28D).

Sample	7D		28D	
	*T*_range_/°C	Mass/%	*T*_range_/°C	Mass/%
GP	14–275	19.9	16–277	21.0
GPSW1	11–258	16.9	12–290	16.6
GPSW2	32–263	18.4	20–253	16.3
GPSW3	27–283	25.1	19–276	23.3
GPSW4	12–285	20.0	24–278	17.5

**Table 5:** Legal limits for disposal in various types of landfills based on the leachate (Directive (EU) 850/2018—Annex 4 of Legislative Decree (Italy) 121/2020).

Parameter	Cr	Ni	Cu	Zn	As	Cd	Pb	Ba	Mo	Sb	Se
Inert	0.05	0.04	0.2	0.4	0.05	0.004	0.05	2.00	0.05	0.006	0.01
Non-Hazardous	1.00	1.00	5.0	5.0	0.20	0.100	1.00	10.0	1.00	0.070	0.05
Hazardous	7.00	4.00	10.0	20.0	2.50	0.500	5.00	30.0	3.00	0.500	0.70

**Table 6:** Comparison data at 28 days of GP aging with wastes added before (GPSW1–4) and after alkali activation (80GP20SW1-4).

Sample	28D					
	pH	IC/mS/m	Mass Loss at 1000 °C/%	σmax/MPa	Leaching Sb/ppm	IHD *E. coli*/cm
GP	10.9	2.8	21.0	22.0	-	1.9
GPSW1	10.9	2.2	32.0	6.9	127.0	1.7
80GP20SW1	7.6	23.1	15.0	68.5	25.0	2.2
GPSW2	11.3	3.3	28.0	3.4	-	2.0
80GP20SW2	10.0	107.6	23.0	37.8	-	2.5
GPSW3	11.3	12.7	29.5	8.0	-	3.2
80GP20SW3	9.8	165.1	13.7	39.4	-	2.5
GPSW4	11.0	3.2	24.8	12.6	0.128	1.9
80GP20SW4	7.9	43.2	12.7	40.4	-	1.9

## Data Availability

The original contributions presented in this study are included in the article. Further inquiries can be directed at the corresponding author.

## Abbreviations

The following abbreviations are used in this manuscript:

MK	Metakaolin
GP	Geopolymer
SW	Solid Waste
SEM	Scanning Electron Microscopy
FT-IR	Fourier-Transform Infrared
TG/DTA	Thermogravimetry–differential thermal analysis
RT	Room Temperature
IC	Ionic Conductivity
IHD	Inhibition Halo Diameter
